# Attempted Experimental Reproduction of Porcine Periweaning-Failure-to-Thrive Syndrome Using Tissue Homogenates

**DOI:** 10.1371/journal.pone.0090065

**Published:** 2014-03-03

**Authors:** Yanyun Huang, John C. S. Harding

**Affiliations:** Department of Large Animal Clinical Sciences, Western College of Veterinary Medicine, University of Saskatchewan, Saskatoon, Saskatchewan, Canada; University of Hong Kong, China

## Abstract

Porcine periweaning failure-to-thrive syndrome (PFTS) is characterized by anorexia and progressive debilitation of newly weaned pigs, of which some also demonstrate repetitive oral behaviour. Although no relevant porcine pathogens have been shown to be causally associated, inoculation of susceptible pigs using tissue homogenates is needed to rule out infectious etiologies. Eight snatched-farrowed porcine-colostrum-deprived (SF-pCD) pigs were inoculated with tissue homogenates made from PFTS-affected pigs orally, or combined orally, intraperitoneally (IP) and intramuscularly (IM) at day (D) 14 of age (INOC). On D21, IP and IM inoculation were repeated. Four sham-inoculated pigs served as control (CTRL). Three INOC pigs developed mixed bacterial septicemia between the first and second inoculation. All other pigs survived until termination on D49. Average daily gain (ADG) and the frequencies of diarrhea did not differ between INOC and CTRL pigs D14 and D29. Additionally, the progressive debilitation characteristic of PFTS was not observed in any pig, and repetitive oral behaviour was observed in both groups. In conclusion, PFTS was not experimentally reproduced by the current experimental approach providing evidence that PFTS may not have an infectious etiology.

## Introduction

Porcine periweaning failure-to-thrive syndrome (PFTS) is typified by newly weaned pigs, apparently healthy at weaning and without residual sickness from the suckling phase, that begin to develop anorexia, lethargy and progressive debilitation within a week after weaning. A portion of affected pigs show repetitive oral behaviour such as chomping and licking, which is regarded as an important characteristic of PFTS [1]. The crude flow prevalence of PFTS in North America was recently estimated to be about 4% [2]. The most frequent lesions of diagnostic relevance are superficial gastritis, small intestinal villous atrophy and thymic atrophy, all of which are observed with higher odds in PFTS-affected versus non-affected animal [3] (and unpublished data). The etiology of PFTS is unknown but infectious causes need to be ruled out. To date, common porcine pathogens, specifically, type 2 porcine circovirus (PCV2), porcine reproductive and respiratory syndrome virus (PRRSV), influenza A virus, transmissible gastroenteritis virus (TGEV) and *Mycoplasma hyopneumoniae* have been conclusively shown not to be associated with PFTS [3], [4] (and unpublished data), whereas hemagglutinating encephalomyelitis virus (HEV), porcine enterovirus CPE groups 1, 2 and 3, rotavirus groups A, B and C, porcine enteric calicivirus (PECV), porcine cytomegalovirus (PCMV) and coccidia (likely *Isospora suis*) may be detected in PFTS pigs but detection is not consistent across cases, and the presence is not associated with clinical status [3], [4] (and unpublished data).

Reproducing the disease in susceptible animals, in this case pigs, is crucial in proving a disease to be of infectious etiology. One of the keys to success is having a reliable animal model using experimental pigs that are immunologically naïve to the presumptive infectious agent(s). Commonly used models for studying swine diseases include the specific pathogen free (SPF), caesarean-derived colostrum-deprived (CDCD) and gnotobiotic models. Each model has advantages and limitations which have been previously reviewed [5]. We have previously developed a snatch-farrowed, porcine colostrum-deprived (SF-pCD) pig model for infectious disease research [5]. SF-pCD pigs experience the advantages of natural birth, are raised on bovine colostrum until weaning at 20 days, can be inoculated during suckling phase and are raised in conventional biocontainment level 2 (BSL2) facilities. Further, SF-pCD pigs are able to mount immune responses similar to conventional pigs but are free of maternally derived antibodies to diseases endemic to the source farm (unpublished data).

The objective of this experiment was to reproduce the clinical signs of PFTS, specifically repetitive oral behaviour and progressive loss of weight and body condition, by inoculating SF-pCD pigs with tissue homogenates derived from pooled organs of PFTS-affected pigs.

## Materials and Methods

### Ethic statement

This work was approved by the University of Saskatchewan's Animal Research Ethics Board and adhered to the Canadian Council on Animal Care guidelines for humane animal use (permit #20110059).

### Experimental procedures

Twelve SF-pCD pigs were born at the Prairie Swine Centre Inc. (Saskatoon, SK, Canada), and raised at the animal care unit (ACU) at the University of Saskatchewan (Saskatoon, SK, Canada) as previously described [5]. Briefly, the pigs were snatched-farrowed, disinfected and placed in HEPA-filtered containers without contacting any farm equipment. Upon arrival at the ACU (day 0), pigs were placed in pens of two, bottle fed for 1–2 days (D), then transitioned as soon as possible to self-feeders until weaning at D21 of age. For the first 21 days, a liquid diet consisting mainly of bovine colostrum was fed. At D21 of age, all pigs were weaned onto an appropriately formulated dry starter diet free of all swine by-products including spray-dried plasma.

On D14, the pens were systematically allocated to 2 inoculated (INOC1, n = 4 pigs; INOC2, n = 4 pigs) and 2 control (CTRL1, n = 2 pigs; CTRL2, n = 2 pigs) groups. The control groups were relocated to a separate BSL2 isolation room and appropriate biosecurity measures implemented to prevent cross contamination between rooms. A 20% w/v tissue homogenate consisting of equal amounts of tonsil, brain, lung, spleen, stomach, small and large intestines collected from 3 PFTS-affected pigs [6] was prepared in minimum essential media (MEM, Life technologies Inc. Burlington, ON, Canada) within a Biological Safety (BSL2) cabinet. The tissues were stored for 8 months at −80°C and thawed immediately before preparation of the homogenate. The homogenate was prepared fresh on each day of inoculation. On D14, INOC1 received 20 ml of the tissue homogenate orally via a gastric tube, while INOC2 received the same dose of homogenate orally as well as 2 ml of 0.2 µm-filtered homogenate both intramuscularly (IM) and intraperitoneally (IP) (Table 1). CTRL1 received 20 ml MEM orally. CTRL2 received 20 ml MEM orally, 2 ml MEM IM and 2 ml MEM IP. On D21, the IM and IP inoculations were re-administered to all pigs.

**Table 1:** Treatment groups and inoculation schedule for PFTS inoculation study.

Groups	Day 14		Day 21	
	Oral	IM+IP	Oral	IM+IP
INOC1 (n = 4)	20 ml non-filtered	NA	NA	2 ml filtered each route
INOC2 (n = 4)	20 ml non-filtered	2 ml filtered each route	NA	2 ml filtered each route
CTRL1 (n = 2)	20 ml MEM	NA	NA	2 ml MEM each route
CTRL2 (n = 2)	20 ml MEM	2 ml MEM each route	NA	2 ml MEM each route

IM = intramuscularly, IP = intraperitoneally, MEM = minimum essential media, NA = Not administered.

To determine the pathogens present in the inocula, a sample of the filtered homogenate was cultured on blood agar aerobically at 37°C overnight. The filtered and non-filtered homogenates were tested for PCV2 [7], PRRSV (Tetracore EZ-PRRSV™ Kit; Rockville, MD, USA), influenza A virus [8], group A rotavirus (unpublished data), HEV [9], TGE [10], PEV_1, 2, and 3 [11], PCMV [12], PECV [13], *Helicobacter-pylori*-like organism and *Helicobacter-heilmannii*-like organism [14] and Mhyo [15] by PCR.

All piglets were monitored twice daily for any clinical sign, and clinical signs of PFTS including repetitive oral behaviour and weight loss in particular. Rectal temperature and body weights were measured daily until D37 of age. Pre-inoculation sera were tested by PCR as previously described [16] to confirm the absence of PCV2 viremia. Pre-inoculation PRRSV testing was not undertaken since the barn of origin was known to be negative.

Piglets were euthanized and necropsied when clinical signs progressed to the point where animal welfare was compromised. Routine aerobic and anaerobic bacterial cultures were performed by Prairie Diagnostic Services (PDS) Inc. (Saskatoon, SK, Canada) on appropriate samples from pigs that developed progressive dyspnea, fever, anorexia and lethargy. All remaining pigs, including controls, were euthanized on D49 of age (35 days after 1^st^ inoculation, 28 days after 2^nd^ inoculation). For all pigs, a thorough necropsy was performed and multiple tissues collected for routine histological examination.

### Statistical analyses

Body weights were compared on D14 (day of first inoculation), D29 (15 days after first and 8 days after second inoculation) and D49 (termination) using Mann Whitney's U test. Linear regression models were used to compare average daily gain (ADG) from D14 to 29 (ADG 14–29), ADG 30–49 and ADG 14–49, while accounting for body weight at the start of the period. All final models were checked for linearity, normality and homoscedasticity of residuals. Pigs euthanized prior to D49 (n = 3) were excluded from the weight and ADG analyses. Fever-days (total days of a pig with rectal temperature ≥40°C) and diarrhea-days (total days of a pig having diarrhea) from D14 to 29 of age were compared between groups by Mann Whitney's U test. Since there were no obvious differences in the frequency of fever and diarrhea, or in body weights between INOC1 and INOC2, CTRL1 and CTRL2, the two INOC groups and two CTRL groups were combined for statistical analyses. Further, because no significant clinical signs were observed after D29, the statistical analyses for fever and diarrhea were only performed on data from D14 to D29. All statistical analyses were performed using IBM SPSS Statistics version 21 (Armonk, NY, USA). *P* values less than 0.05 were regarded as statistically significant and values between 0.05 and 0.1 were considered indicative of a trend.

## Results

The filtered and non-filtered homogenates tested negative for PCV2, TGE, influenza A virus, PRRSV, group A rotavirus, PECV, Mhyo and *Helicobacter-pylori*-like organism, while positive for PCMV, PEV CPE groups 1, 2 and 3. *Helicobacter-heilmannii*-like organism was detected in the non-filtered but not the filtered homogenate. There was no bacteria growth from the filtered homogenate under aerobic condition.

All pigs grew at acceptable rates and appeared in good body condition before the first inoculation at D14 of age. One pig in INOC1 (oral only) and two pigs in INOC2 (oral+IM+IP) developed progressive dyspnea, fever, anorexia and lethargy after the 1^st^ inoculation and were humanely euthanized before the 2^nd^ inoculation. The first evidence of illness began on D15, D15 and D17, respectively, in these three pigs, which were euthanized on D18, D16 and D20 of age, respectively. Postmortem and histological examination revealed bronchopneumonia (2/3), pericarditis (3/3), pleuritis (3/3), and peritonitis (2/3) associated with mixed bacterial infections of lung, spleen and synovium with *E. coli*, *Streptococcus suis*, *Fusobacterium* spp. and *Staphylococcus* spp. Two additional INOC1 and two INOC2 pigs developed transient fever of 1 to 2 days duration between the 1^st^ and 2^nd^ inoculation but remained otherwise healthy. One pig in each CTRL group developed fever for 1 day each, on D23 and D20, respectively, but also remained otherwise healthy. When all pigs, including those euthanized, were included in the analysis, the number of fever-days was not significantly different between INOC and CTRL (*P* = 0.37) during the 2-week period following the first inoculation. No INOC pigs developed illness or fever after the 2^nd^ inoculation.

Between D14 and D29, transient diarrhea characterized by small to moderate amounts of watery feces for 1 to 2 days developed in all but one INOC pig. Diarrhea was also observed in 3/4 CTRL pigs during this period. The number of diarrhea-days was not significantly different between INOC and CTRL (*P* = 0.37) during the 2 week period following the first inoculation. No diarrhea was noted following the second inoculation.

Body weights of surviving animals did not differ between group on D14, D29 or D49 (Table 2). The ADG 14_29 of surviving pigs trended higher in CTRL than INOC, and ADG 29_49 was significantly higher in INOC than CTRL (Table 2). Body weight at D29 was positively related with ADG 29_49 (*P* = 0.001, beta = 0.044 kg/d). ADG14_49 did not differ between group (Table 2).

**Table 2:** Median body weight (kg) and average daily gain (ADG; kg/d) at selected time points following inoculation at day 14 and 21.

	INOC* (IQR)†	CTRL (IQR)	*P*
Weight D14 (pre-inoculation)	3.1 (1.4)	2.5 (1.1)	ns‡
Weight D29 (14 days post inoculation 1)	5.8 (2.2)	5.6 (3.0)	ns
Weight D49 (termination)	15.5 (4.3)	13.6 (5.8)	ns
ADG D14 to D29	0.17 (0.07)	0.20 (0.13)	0.1
ADG D29 to D49	0.49 (0.10)	0.40 (0.16)	0.03
ADG D14 to D49	0.35 (0.06)	0.32 (0.13)	ns

*INOC, n = 5; CTRL, n = 4; Euthanized (septicemic) pigs were excluded from analyses.

† IQR = Interquartile range.

‡ ns = not significant.

All pigs regardless of treatment showed repetitive chomping and licking behaviour typical of PFTS after weaning. This oral behaviour was noted for a brief period of time from 24 to 48 hours after abrupt weaning from liquid diet on day 21, and before the pigs learned to eat dry starter diet from the feeder. The behaviour ceased as soon as solid feed was consumed.

## Discussion

The current study represents the first attempt to experimentally reproduce PFTS using tissue homogenates from PFTS-affected pigs and the SF-pCD model. Although repetitive oral behaviour (chomping) was observed in both groups, it is clear that the current approach failed to reproduce the progressive loss of body weight and condition within 2 weeks of weaning that is characteristic of the syndrome. Nevertheless, the results of this study provide important and novel insights.

The inoculation strategy of this experiment aimed to maximize the likelihood of reproducing PFTS. Firstly, two inoculations were performed; one week before and on the day of weaning. Though typical clinical signs of PFTS are by definition observed shortly after weaning, it is possible that if caused by infective agent(s), the initial exposure occurs during the suckling phase. For this reason, the first inoculation was performed before weaning. The second inoculation was on the weaning day, when pigs experience stresses associated with a change of diet and reduction in oral immunoglobulin consumption. Secondly, the combination of different inoculation routes mimicked both gastrointestinal and systemic exposure. Although the most frequently observed lesions of PFTS suggest the gastrointestinal tract to be a primary organ of pathogenesis [3] (and unpublished data), it is also possible that a systemic infection causes anorexia which then induces secondary lesions of lymphocytic gastritis and small intestinal villus atrophy. The application of oral, IM and IP inoculation was an attempt to cover these possible pathogeneses. Thirdly, the combination of multiple tissues in the homogenate accounted for the possibility that the etiology of PFTS might dwell in non-gastrointestinal tissues such as brain, lung and spleen. Using multiple tissues in the inocula however, increased the risk of diluting a presumptive infectious agent if that agent was localized in some but not other tissues. With these uncertainties in mind, our inoculation strategy for this first attempt was designed to ensure early and broad exposure. Following the 1^st^ inoculation it was clear that oral inoculation of non-filtered homogenate caused bacterial septicemia which is not a feature of PFTS. It was for this reason that the 2^nd^ inoculation used only filtered inoculum. MEM was selected as the inoculum for control groups because our goal was to ensure clinical outcomes were as divergent as possible. Had PFTS been experimentally reproduced herein, additional experiment(s) using tissue inocula collected from PFTS-affected and healthy control pigs would have been undertaken.

The failure to reproduce progressive loss of weight and body condition characteristic of PFTS in this experiment suggests the etiology of PFTS may not be infectious, although a more definite conclusion cannot be made based on a single experiment. Although less likely, it also cannot be definitely ruled out that the causative agent of PFTS (if any) in the inoculum was not in a high enough concentration to cause clinical sickness.

It was clear that the clinical signs in the three INOC pigs euthanized following first inoculation were associated with septicemia of mixed bacterial origin, demonstrating that the SF-pCD pigs were susceptible to “opportunistic” bacterial pathogens. These bacteria were likely derived from the PFTS-affected pigs used to derive the tissue inocula. On the other hand, we cannot rule out the possibility that the tissue inocula also contained bacteria from the necropsy facility when the PFTS pigs were necropsied. Regardless of the origin of these bacteria, the polyserositis observed in these 3 INOC pigs was not consistent with PFTS (polyserositis is not a primary lesion of PFTS) [3].

A number of other potential pig pathogens were retrospectively identified in the inoculum including PCMV, PEV and *Helicobacter-heilmannii*-like organism. In spite of this, no histological findings consistent with *Helicobacter-heilmannii*-like organism (also known as *Candidatus Helicobacter suis*) [17], PEV [11] or PCMV were observed indicating these potential pathogens failed to induce characteristic lesions or disease in this experimental model. In agreement with other studies [6] (and unpublished data), these data provide additional evidence that these organisms are not the cause of PFTS.

Most INOC pigs developed diarrhea and fever between D14 and D29. However, diarrhea and fever was also observed in some CTRL pigs that remained otherwise healthy, and the diarrhea-days and fever-days were not significantly different between INOC and CTRL groups. In our experience, pre-weaning diarrhea is frequently observed in SF-pCD pigs and although the mechanism is not fully understood, the diarrhea resolves soon after weaning. The diarrhea in CTRL therefore was not unexpected, and the diarrhea observed in the INOC pigs may be a combination of “physiological”, nutritional and pathological diarrhea.

An interesting finding in this experiment was that repetitive oral behaviour (chewing and chomping) was observed in all pigs (INOC and CTRL) shortly after weaning and before the pigs ate solid feed. Although the presence of excessive repetitive oral behaviour is clearly associated with PFTS, chomping was obviously not induced by inoculation in this study. This observation led to our suspicion that chomping may be a behaviour associated with hunger or abdominal discomfort. Indeed, when sows are feed restricted, repetitive sham chewing is a well recognized stereotypic behaviour [18]. Moreover, our group has also documented repetitive oral behaviour in a small proportion of commercial nursery pigs one and 4 weeks post weaning in the absence any obvious disease (unpublished data). Collectively, these findings indicate that the repetitive oral behaviour is not specific to PFTS.

A recent experiment conducted at the University of Minnesota attempted to reproduce PFTS by inoculating pigs with HEV, rotavirus group A, or a combination of HEV, rotavirus group A and PRRSV. It was reported that clinical signs of PFTS were observed in all inoculation groups as well as in a sham-control group [19]. Unfortunately, the observed clinical signs were not specified, nor did body weights differ among group. It is obvious that this experiment also did not reproduce progressive loss of weight and body condition, which is an important feature of PFTS and a fundamental part of the clinical case definition [20]. Further, no histological changes characteristic of PFTS [6] were observed in the experiment [19]. Thus, one should be cautious when citing that PFTS has been experimentally reproduced.

The current experiment also serves to verify that SF-pCD pig is a valid model for study of infectious diseases. Although specific pathogen free (SPF), Caesarian-derived, colostrum-deprived (CDCD) and gnotobiotic pig models are commonly used for swine infectious disease studies, these models have disadvantages. SPF pigs are typically conventional pigs that have waned maternal antibodies after weaning. Despite its convenience and economical nature, one cannot obtain younger SPF pigs. Thus, this model is not suitable to study the effect of pathogens on suckling pigs, especially when the pathogen of interest is prevalent making it difficult to locate a negative farm. CDCD and gnotobiotic pigs do not experience natural birth. It is well documented that before natural birth, pigs and other livestock species experience a pre-parturient cortisol surge [21] that is important for tissue maturation, immunoglobulin absorption and glycogen deposition in muscle and liver [22]. This may explain why Caesarean-derived pigs typically have higher mortality than naturally delivered pigs even if delivered at term. An additional drawback of gnotobiotic pig is that they possess a distorted immune response because they lack bacterial colonization in the gut [23]. This indicates the gnotobiotic model is not always a satisfactory model although it has undoubtedly served as a powerful tool for swine infectious disease research in the past.

The development of the SF-pCD model addresses the weaknesses of other swine models. Previous efforts to raise SF-pCD pigs by other researchers obtained 80% or less survival rate [24], [25]. After some modification, we have consistently raised (non-inoculated) SF-pCD pigs with 100% survival [5]. Further, it has been shown that SF-pCD pigs were able to mount an immune response similar to that of conventional pigs (unpublished data). The use of SF-pCD pigs in this present experiment, despite the failure of reproducing the body weight loss associated with PFTS, demonstrates that these pigs are susceptible to systemic bacterial infection. This is noteworthy, since bovine colostrum, in addition to containing immunoglobulins G_1_, G_2_, M and A, contains a variety of minor constituents and peptides with innate immunological activity such as neutrophils, macrophages, complement, cytokines, acute phase proteins and others [26]. The degree to which these confer protection to the pig at the local or systemic level is unknown, but these constituents presumably provide some protection against environmental and opportunistic pathogens. It appears that they do not however, protect against overwhelming bacterial challenge. Although susceptibility to viral pathogens has not yet been demonstrated, SF-pCD pigs are presumably susceptible to infective doses typical observed in natural or experimental settings.

In conclusion, the progressive loss of body weight and condition, a key feature of PFTS and part of the clinical case definition, was not reproduced following the inoculation of SF-pCD pigs with tissue homogenates derived from PFTS-affected pigs. This study therefore provides evidence that PFTS may not be caused by an infectious etiology.
